# Multi-timescale phase-amplitude couplings in transitions of anesthetic-induced unconsciousness

**DOI:** 10.1038/s41598-019-44238-8

**Published:** 2019-05-24

**Authors:** Feng-Fang Tsai, Shou-Zen Fan, Hsiao-Liang Cheng, Jia-Rong Yeh

**Affiliations:** 1 Department of Anaesthesiology, National Taiwan University Hospital, Taipei, Taiwan; 2 Department of Anaesthesiology, College of Medicine, National Taiwan University, Taipei, Taiwan; 3 Graduate Institute of Clinical Medicine, College of Medicine, National Taiwan University, Taipei, Taiwan; 4 Research Center for Adaptive Data Analysis, National Central University, Taoyuan, Taiwan; 5 Center for Nonlinear Sciences, Pilot National Laboratory for Marine Science and Technology, Qingdao, China

**Keywords:** Electroencephalography - EEG, Biomedical engineering, Computational science

## Abstract

Under general anesthesia (GA), advanced analysis methods enhance the awareness of the electroencephalography (EEG) signature of transitions from consciousness to unconsciousness. For nonlinear and nonstationary signals, empirical mode decomposition (EMD) works as a dyadic filter bank to reserve local dynamical properties in decomposed components. Moreover, cross-frequency phase-amplitude coupling analysis illustrates that the coupling between the phase of low-frequency components and the amplitude of high-frequency components is correlated with the brain functions of sensory detection, working memory, consciousness, and attentional selection. To improve the functions of phase-amplitude coupling analysis, we utilized a multi-timescale approach based on EMD to assess changes in brain functions in anesthetic-induced unconsciousness using a measure of phase-amplitude coupling. Two groups of patients received two different anesthetic recipes (with or without ketamine) during the induction period of GA. Long-term (low-frequency) coupling represented a common transitional process of brain functions from consciousness to unconsciousness with a decay trend in both groups. By contrast, short-term coupling reflected a reverse trend to long-term coupling. However, the measures of short-term coupling also reflected a higher degree of coupling for the group with ketamine compared with that without ketamine. In addition, the coupling phase is a factor of interest. The phases for different combinations of coupling components showed significant changes in anesthetic-induced unconsciousness. The coupling between the delta-band phase and the theta-band amplitude changed from in-phase to out-phase coupling during the induction process from consciousness to unconsciousness. The changes in the coupling phase in EEG signals were abrupt and sensitive in anesthetic-induced unconsciousness.

## Introduction

Under general anesthesia (GA), understanding changes occurring in the dynamical properties of electroencephalography (EEG) from consciousness to unconsciousness is a crucial topic in the field of anesthesiology. Traditional spectral analysis provides simple conclusions, such that the effectiveness of GA is associated with an increase in the spectral power of surface EEG at frequencies less than 40 Hz^1^. However, the neuronal oscillations of different frequency bands as EEG components can interact with one another^2^. The interaction between the oscillatory components of different frequency bands in EEG is called cross-frequency coupling (CFC). In many studies, a decrease in low-frequency CFC is considered an anesthetic-induced change in EEG^3–5^. Phase-amplitude coupling (PAC) is a typical method used to assess CFC, which reflects the coupling between the phase of low-frequency rhythms and the amplitude of high-frequency oscillations. For example, the theta–gamma PAC in the hippocampus is involved in the organization of sequential memory and the maintenance of working memory^6–8^. Moreover, the measure of PAC is associated with sensory detection^9^, attentional selection^10^, and memory processes^11,12^. In applications of anesthesiology, the phase-based coupling measure has been used to investigate anesthetic-induced unconsciousness^13–16^. Coupling changes occurred between the phase of low-frequency rhythms and the amplitude of alpha-band oscillations (8–14 Hz) in EEG recordings during sevoflurane infusion^16^. Therefore, CFC analysis can be utilized to effectively investigate the underlying mechanism in GA. Furthermore, investigations on the functional connectivity of the brain network can be explored using PAC analysis in a scenario using whole-brain surface EEG recordings^17–20^. Moreover, improving the performance of PAC analysis benefits further investigations of functional connectivity in the human brain.

Tort *et al*. suggested the modulation index (MI) to detect PAC between two frequency bands of interest: the “phase-modulating” and “amplitude-modulated” frequency bands^21^. The measure of the MI is defined as an adaptation of the Kullback–Leibler (KL) distance, which is a function used to infer the distance between two distributions^22,23^. This measure reflects how much an empirical amplitude distribution-like function over phase bins deviates from the uniform distribution. The plot of the amplitude distribution-like function over phase represents the information of the coupling phase.

Thus far, most applications of PAC analysis have extracted the components of frequency bands of interest using linear band-pass filters. In this study, empirical mode decomposition (EMD) is considered a superior choice for decomposing nonlinear and nonstationary signals, such as surface EEG signals, into a set of intrinsic mode functions (IMFs)^24,25^. EMD works as a dyadic filter bank to extract intrinsic components adaptively^26^. Therefore, predetermining the frequency bands of EEG signals is not necessary. The intrinsic frequency bands of mode functions are determined adaptively to the nature of signals. Therefore, multi-timescale PAC analysis can be conducted on multiple pairs of phases of low-frequency IMFs and amplitudes of high-frequency IMFs systematically and automatically. The PAC measures and the coupling phase are two properties derived for each pair of coupling to represent the coupling characteristics of EEG in GA.

In a clinical experimental design, ketamine selectively bound to N-methyl-D-aspartate receptors on the GABAergic inhibitory interneurons of the pyramidal system and caused a disinhibition reaction^27^. The use of ketamine significantly increased the value of the bispectral index (BIS) in sevoflurane-induced GA^28^. Thus, we sought to answer the following question: what differences will be stimulated when ketamine is used as an integrant of an anesthetic recipe? For our control case, only alfentanil was used in GA. We hypothesized that the results of multi-timescale PAC analysis would sufficiently represent the common characteristics in coupling for anesthetic-induced unconsciousness as well as the particular changes caused by ketamine. A total of 60 patients with low anesthetic risk (ASA I-II) were recruited into this study. Sevoflurane with inhalation induction was used to induce patients into sedative unconscious states. Patients received different regimens when losing consciousness, which were confirmed by an anesthesiologist. The brain state during the first 20 minutes of GA can be roughly divided into three states of awareness: baseline, sevoflurane-induced unconsciousness, and anesthetic-induced unconsciousness. Both the MI and coupling phase for multi-timescale PAC analysis were used to investigate the dynamics of surface EEG in GA.

## Results

### Intrinsic frequency bands for components were decomposed by EMD

**Figure 1:**
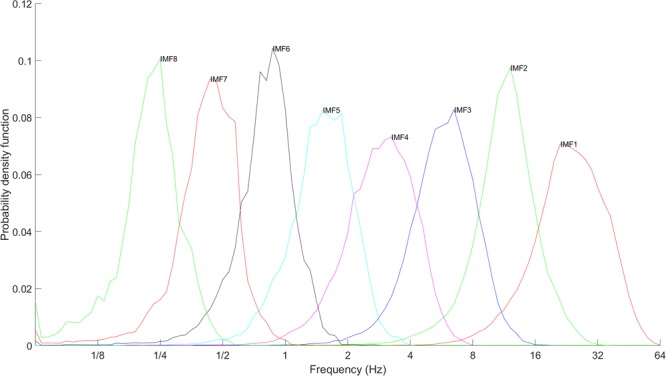
Distributions of intrinsic frequency bands for IMFs decomposed from EEG signals. The sampling rate of EEG signals is 128 Hz. Since EMD functions as a dyadic filter bank, the X-axis of frequency is shown in logarithmic scale, and the Y-axis represents the probability density function.

In this study, an EEG signal was decomposed into a set of IMFs by EMD. Because decomposition is adaptive to the nature of EEG signals, the frequency band for each IMF is not predetermined. We derived the time series of instantaneous frequency (IF) and amplitude for each IMF using Hilbert transformation, and the range of the instantaneous frequency distribution represented the intrinsic frequency band of an IMF. To map the intrinsic frequency bands of IMFs decomposed from EEG signals onto the traditional frequency bands in spectral analysis, we plotted the distributions of instantaneous frequency (Fig. 1) for IMFs decomposed from EEG recordings, which were recorded at a sampling rate of 128 Hz. The intrinsic frequency bands satisfied the condition of dyadic interaction in its most essential respects. The medians of intrinsic frequency bands for the first seven IMFs are shown in Table 1. Because the waveforms of IMFs are nonlinear and nonstationary, the logarithmic distributions of IF are wider than those of dyadic frequency bands. Intrawave and interwave frequency modulations can be observed in the time series of IF. The ranges of intrinsic frequency bands were determined using 70% of the logarithmic distributions of IF, which represents an appropriate boundary between the IF distributions of two successive IMFs. According to intrinsic frequency bands shown in Table 1, the highest intrinsic frequency band is similar to the beta band in traditional spectral analysis. Three IMFs (IMFs 5–7) have intrinsic frequency bands located on the delta band (approximately 0.5–4 Hz). An IMF with an intrinsic frequency band similar to a gamma band cannot be decomposed due to poor sampling resolution.

**Table 1:** Medians and ranges of instantaneous frequency bands of IMFs decomposed from EEG signals at a sampling rate of 128 Hz.

IMF	Median of IF	Range of IF	Mapped frequency band
1	24.32 Hz	14.9~34.3 Hz	Beta
2	11.72 Hz	8.0~14.9 Hz	Alpha
3	6.00 Hz	4.0~8.0 Hz	Theta
4	2.98 Hz	1.87~4.00 Hz	High delta
5	1.53 Hz	1.00~2.00 Hz	Middle delta
6	0.83 Hz	0.57~1.07 Hz	Low delta
7	0.44 Hz	0.29~0.53 Hz	Less than delta

### Multi-timescale phase-amplitude plots for CFCs

As mentioned previously, decomposition by EMD is adaptive to the nature of a signal, in which the intrinsic frequency bands of IMFs are not predetermined. The intrinsic frequency bands of IMFs can be mapped onto similar frequency bands defined in traditional spectral analysis; however, the bandwidth of intrinsic frequency bands does not match that of traditional frequency bands defined for the EEG spectrum. Therefore, the coupling between the phase of IMF2 and the amplitude of IMF1 is more appropriate than the coupling between the alpha-band phase and beta-band amplitude in the EMD-based approach of PAC analysis.

**Figure 2:**
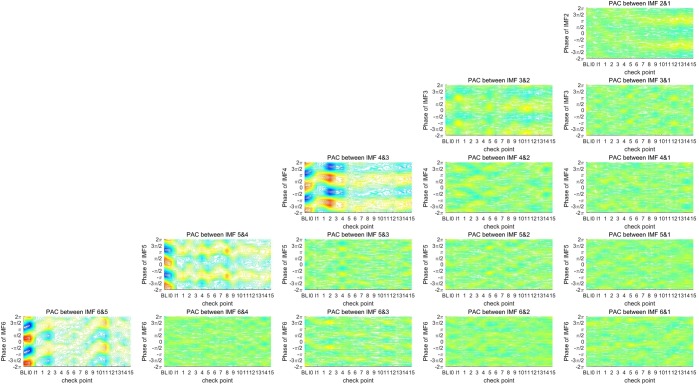
Phase-amplitude plots for 15 PAC couplings at 18 checkpoints for Group A, who received a mixture of ketamine and alfentanil. The amplitude of high-frequency IMF is shown in different colors; Y-axis represents the phase of low-frequency IMF. Each sub-figure profiles the phase-amplitude plots covering 18 checkpoints for each PAC coupling.

**Figure 3:**
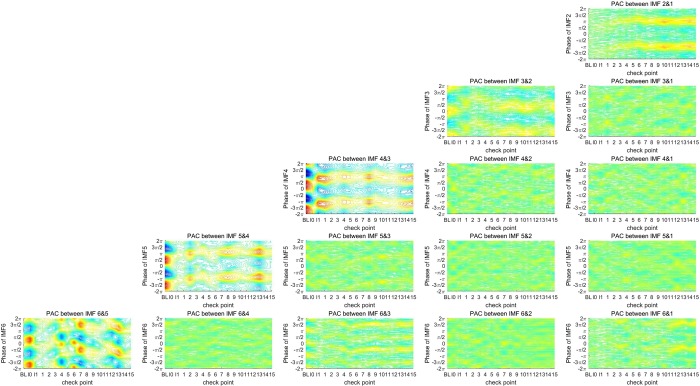
Phase-amplitude plots for 15 PAC couplings at 18 checkpoints for Group B, who received only alfentanil; the amplitude of the high-frequency IMF is shown in different colors; Y-axis represents the phase of the low-frequency IMF. Each sub-figure profiles the phase-amplitude plots covering 18 checkpoints for each PAC coupling.

As shown in Table 1, because the intrinsic frequency bands of the first six IMFs cover the most defined frequency bands of the EEG spectrum, a total of 15 PAC couplings exist between the phases of low-frequency IMFs and the amplitudes of high-frequency IMFs, including the phase of IMF2 that couples exclusively with an amplitude of IMF1, the phase of IMF3 that couples with amplitudes of IMFs 1 and 2, and the phase of IMF6 that couples with amplitudes of the first five IMFs. Moreover, we defined 18 checkpoints for observing changes in PAC coupling across the time period of anesthetic induction. The first checkpoint is the period before induction, which is termed the baseline and denoted as “BL”. The second checkpoint is the start time point of sedation-induction using sevoflurane, which is denoted as “I0”. The third checkpoint is the time of losing consciousness confirmed by an anesthesiologist, which is denoted as “I1”, and the checkpoints for the first 15 minutes after the patients received various anesthetic agents, which are denoted by numbers. For GA, the patients in Group A received a mixture of ketamine with alfentanil (experimental group), and those in Group B received only alfentanil (control group). Figure 2 shows the averaged phase-amplitude plots of 15 couplings for Group A (experimental), and Fig. 3 shows the averaged phase-amplitude plots for Group B (control).

EEG signals for the first three checkpoints (baseline, I0, and I1) were recorded under similar conditions for the two groups. According to the results of the phase-amplitude plots shown in Figs 2 and 3, only the five couplings of successive IMFs (between the phase of IMF2 and the amplitude of IMF1, the phase of IMF3 and the amplitude of IMF2, and so on) represent significant PAC couplings, in which the distribution-like function of the amplitude is significantly different from a uniform distribution. Notably, the phase-amplitude plot of the coupling between the phase of IMF4 and the amplitude of IMF3 represents a dramatic change from consciousness to sedation-induced unconsciousness. The changes are abrupt between the I0 and I1 checkpoints, representing the change in the brain state from consciousness to unconsciousness. The distribution-like function of the amplitude over phase becomes gradually nonsignificant, and the coupling phase is shifted. In addition, changes in coupling between the phase of IMF2 and the amplitude of IMF3 are noteworthy. During the time period covering the baseline and first 2 minutes after receiving anesthetic agents, the phase-amplitude plots of coupling between IMFs 1 and 2 represent nonsignificant coupling because the amplitude distribution-like function is similar to a uniform distribution. At the checkpoint of the fifth minute after receiving anesthetic agents, the coupling between the phase of IMF1 and the amplitude of IMF2 gradually becomes significant on the phases around π. The increasing trend of coupling between the phase of IMF1 and the amplitude of IMF2 for the control group is more significant than that for the experimental group. Thus, ketamine appears to suppress the gradually increasing trend of coupling between the phase of IMF1 and the amplitude of IMF2 in GA. The phase-amplitude plots present some interesting results through graphic presentations, and it would be helpful to represent the results of PAC coupling using quantitative indicators. The statistical analysis helps to prove the differences in the brain state caused by ketamine in GA.

### Results of the MI for multiple couplings

In this study, multiple PAC couplings among the first six IMFs were quantified using a simple quantitative indicator, the MI. The MI represents how much the amplitude distribution-like function over the phase deviates from a uniform distribution. A uniform distribution represents a totally uncoupled relationship between the phase of low-frequency components and the amplitude of high-frequency components. According to the results shown in Figs 2 and 3, two different states occur after receiving anesthetic agents. The first state is 5 minutes after receiving anesthetic agents, which is an unstable state, and the brain state then gradually stabilizes. Therefore, the 18 checkpoints were divided into four stages for further comparison between the responses of the two groups under GA. The first stage is the baseline, which represents the state before anesthesia. The second stage is the sedation state, which represents sedative-induced unconsciousness using sevoflurane. The third stage covers the first 5 minutes after receiving anesthetic agents, which is denoted as “min 1–5”. The last stage represents a gradually stable stage in GA, which is denoted as “anesthesia.”

To identify the interesting PAC couplings correlated to the transition of consciousness in anesthesia, analysis of variance (ANOVA) was used for intra-group comparisons of different stages. The ANOVA results present the statistical differences among the four stages (baseline, induction, the first 5 minutes in anesthesia, and GA) for MI values of 15 PAC couplings. According the statistical results shown in Table 2, the PAC couplings between two successive IMFs and the PAC couplings between phases of IMF4–5 and the amplitude of IMF3 (the frequency band similar to the theta band) show statistical differences among 4 stages. These results serve as a guide for identifying the interesting PAC couplings, which reflect transitions in consciousness from aware to anesthetic-inducted unconsciousness.

**Table 2:** The statistical results (ANOVA) for 15 PAC couplings for intra-group comparisons among 4 stages.

ID	PAC coupling between	p-value for group A	p-value for group B
1	Phase of IMF2 & amplitude of IMF1	0.0053	0.0000
2	Phase of IMF3 & amplitude of IMF1	0.5151	0.2148
3	Phase of IMF4 & amplitude of IMF1	0.2717	0.9661
4	Phase of IMF5 & amplitude of IMF1	0.1940	0.1900
5	Phase of IMF6 & amplitude of IMF1	0.4913	0.0253
6	Phase of IMF3 & amplitude of IMF2	0.0005	0.0027
7	Phase of IMF4 & amplitude of IMF2	0.0217	0.0002
8	Phase of IMF5 & amplitude of IMF2	0.0001	0.0000
9	Phase of IMF6 & amplitude of IMF2	0.0009	0.0071
10	Phase of IMF4 & amplitude of IMF3	0.0000	0.0000
11	Phase of IMF5 & amplitude of IMF3	0.0000	0.0000
12	Phase of IMF6 & amplitude of IMF3	0.0000	0.0000
13	Phase of IMF5 & amplitude of IMF4	0.0000	0.0000
14	Phase of IMF6 & amplitude of IMF4	0.6199	0.4255
15	Phase of IMF6 & amplitude of IMF5	0.0000	0.0648

**Figure 4:**
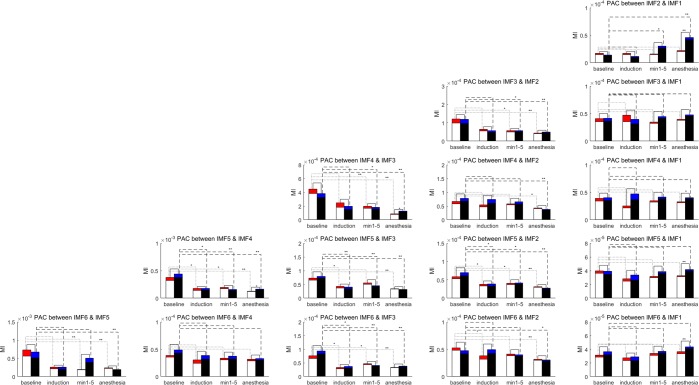
Statistical results of modulation indexes for 15 PAC couplings for two groups and four stages. Means and standard deviations of modulation are shown as the bar charts for two groups in 4 stages. The solid lines represent the statistical difference between two groups in the same stage. Dotted lines represent the statistical differences between MI values for 3 unconscious stages compared with those for baseline for group A. Dash lines represent those for group B. For each line, ‘*’ represents a significant difference with a p-value of <0.01, and ‘**’ represents a significant difference with a p-value of <0.001.

Moreover, paired KS-tests were used to determine the statistical differences between two groups in the same stage and between baseline and the other stages for the same group. MI values for the two groups and four stages are presented using means and standard deviations. Figure 4 presents the statistical results for 15 PAC couplings. According to the study on PAC measurement conducted by Tort *et al*., data length is a critical factor for calculating the MI. Longer epochs lead to smaller variations in MI, and a higher intensity of coupling is associated with lower variations. Moreover, strong coupling can be more confidently inferred than weak coupling. Because the values of the MI are shown, the five PAC couplings between coupling pairs of two successive IMFs, excluding the coupling between the phase of IMF2 and the amplitude of IMF1, present strong coupling, with a higher MI value between 0.0001 and 0.001. The other couplings are significantly weak, with MI values less than 0.0001. Therefore, the statistical results for the five couplings between the coupling pairs of two successive IMFs are discussed in this section.

The p-values between the values of the MI in the anesthesia stage for the two groups (with/without ketamine) are significant for the couplings between the phase of IMF2 and the amplitude of IMF1 (p < 0.001 using the Kolmogorov–Smirnov (KS) test), the phase of IMF4 and the amplitude of IMF3 (p < 0.01 by KS), and the phase of IMF5 and the amplitude of IMF4 (p < 0.01 by KS). The MI values for Group A are lower than those for Group B without ketamine, which implies that ketamine suppresses the gradually increasing trend of PAC coupling on these three corresponding frequency bands correlated with the intrinsic timescales of IMFs.

The statistical differences between the baseline and min 1–5 are significant for the couplings between the phase of IMF3 and the amplitude of IMF2, the phase of IMF4 and the amplitude of IMF3, and the phase of IMF5 and the amplitude of IMF4 (p < 0.01 by KS). Furthermore, the statistical differences between the baseline and stage of anesthesia are significant for the couplings between the phase of IMF3 and the amplitude of IMF2, the phase of IMF4 and the amplitude of IMF3, and the phase of IMF5 and the amplitude of IMF4 (p < 0.001 by KS).

The MI results proved that the decreases in PAC couplings covering the intrinsic timescales for the phases of IMFs 3–5 and the amplitudes of IMFs 2–4 are associated with anesthetic-induced unconsciousness. Moreover, ketamine actually suppressed the increasing trend of coupling between the phase of IMF2 and the amplitude of IMF1 under GA.

### Shifting of the coupling phase in sevoflurane-induced unconsciousness and anesthesia

**Figure 5:**
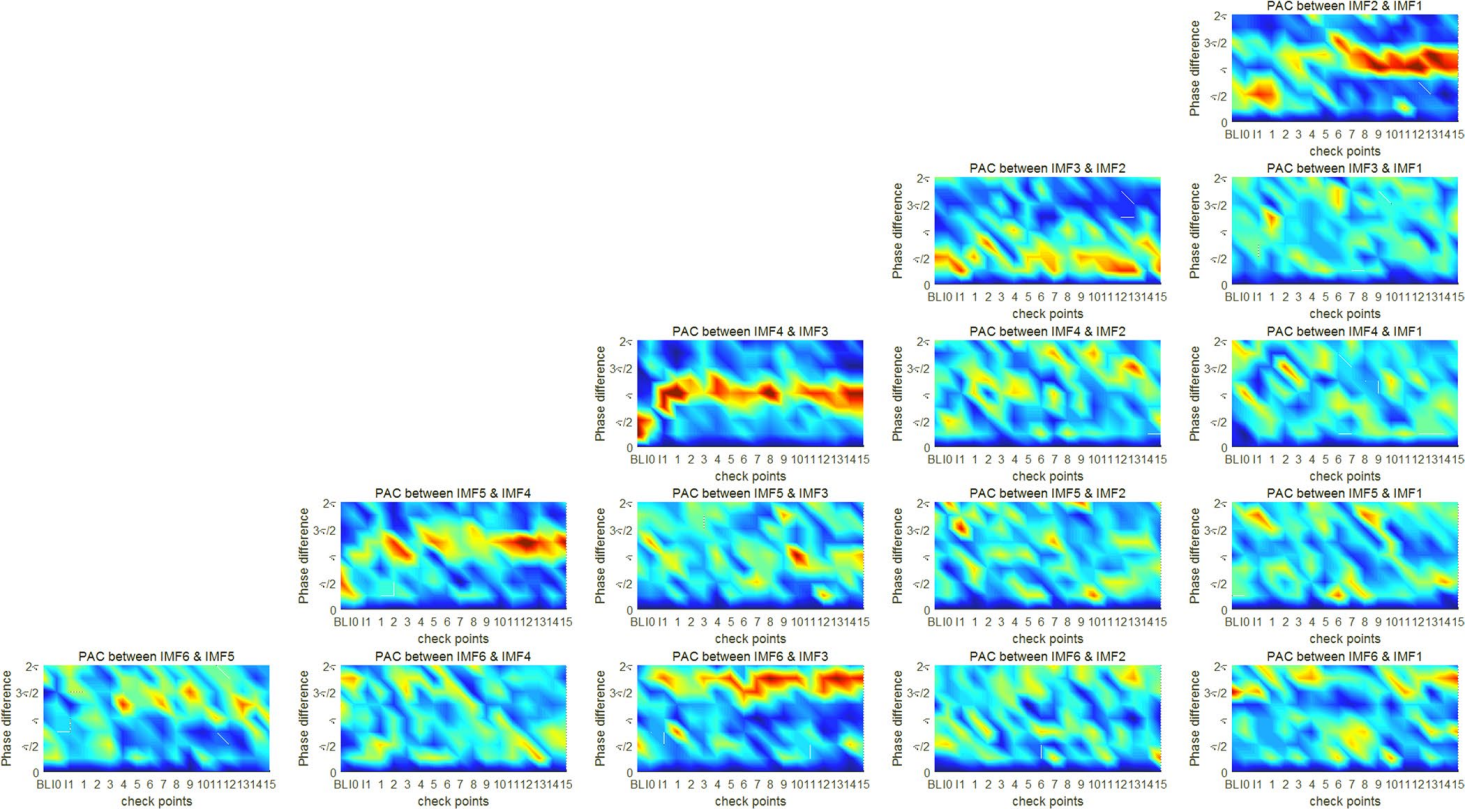
Probability density functions of the coupling phase for 15 couplings over multiple intrinsic timescales for Group A. Each sub-figure shows the distributions of the coupling phase for 18 checkpoints. The domain of the coupling phase was divided into 16 bins for calculating the probability density function for each bin, and the distribution of the coupling phase is shown with probability density functions over 16 bins.

In this study, the coupling phase was defined as the phase of the peak for an empirical amplitude distribution-like function over the phase. For a strong coupling, the coupling phase is clear and easy to determine. However, the coupling phase is ambiguous for a weak coupling. Figure 5 shows the probability density functions of the coupling phase for all the checkpoints and 15 PAC couplings. The difference in the coupling phase between the I0 and I1 checkpoints represents the change in the coupling phase caused by sevoflurane-induced unconsciousness. The change in the coupling phase can be clearly identified in the phase-amplitude plot for the coupling between the phase of IMF4 and the amplitude of IMF3. The peak of the probability density function of the coupling phase is located on the phase of π/2 at checkpoint I0, and it is shifted to the phase of π at the checkpoint of I1.

The coupling phase for the coupling between the phase of IMF2 and the amplitude of IMF1 is concentrated on the phase around π for checkpoints from the sixth to fifteenth minute after receiving anesthetic agents. According to the phase-amplitude plots for the coupling between the phase of IMF2 and the amplitude of IMF1 (Figs 2 and 3), PAC couplings are weak before the sixth minute checkpoint after receiving anesthetic agents. Because the coupling phase is ambiguous for a weak PAC coupling, a coupling phase around π for the coupling between the phase of IMF2 and the amplitude of IMF1 is a critical characteristic of a stable state under GA.

**Figure 6:**
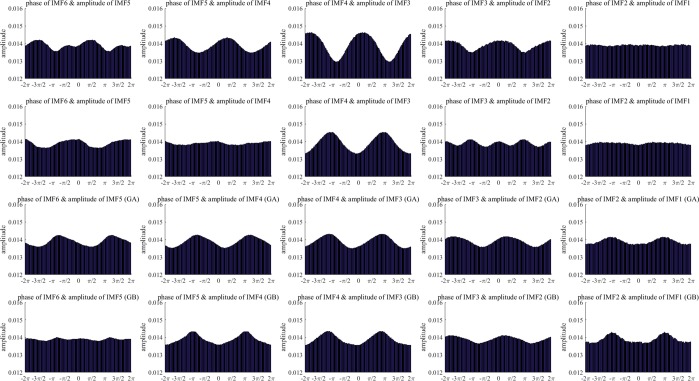
Phase-amplitude plots for five PAC couplings between pairs of two successive IMFs. The top row represents the couplings at baseline. The second row represents the couplings in sevoflurane-induced unconsciousness. The third and fourth rows represent the couplings in anesthetic-induced unconsciousness for Groups A and B, respectively.

To demonstrate the characteristics of coupling phases for different stages, Fig. 6 shows the averaged phase-amplitude plots for the two groups at three stages. The phase-amplitude plots for five PAC couplings between the pairs of two successive IMFs represent the phases of low- and high-frequency components on their corresponding intrinsic timescales. From a long to a short timescale, the five PAC couplings are from the phase of IMF6 and the amplitude of IMF5, the phase of IMF5 and the amplitude of IMF4, and the phase of IMf2 and the amplitude of IMF1. As shown in Fig. 6, each row contains five phase-amplitude plots from long to short timescales. Four rows of phase-amplitude plots represent the different brain states at baseline, sevoflurane-induced unconsciousness, and anesthetic-induced (with ketamine) unconsciousness for Group A and anesthetic-induced (without ketamine) unconsciousness for Group B.

The five phase-amplitude plots for the baseline show that significant PAC couplings can be observed among the intrinsic frequency bands covered by IMF2–IMF6. The strongest PAC coupling is that between the phase of IMF4 and the amplitude of IMF3; its coupling phase is located around the phase of zero. The second row of plots represents five phase-amplitude plots for sevoflurane-induced unconsciousness. The most significant change in PAC coupling from consciousness to sevoflurane-induced unconsciousness is the shifting of the coupling phase from 0 to π and the coupling between the phase of IMF4 and the amplitude of IMF3. The amplitude distribution of the coupling between the phase of IMF3 and the amplitude of IMf2 exhibits a multimodal distribution, and the amplitude distribution of the coupling between the phase of IMF5 and the amplitude of IMF4 is similar to a uniform distribution, which represents a weak coupling, in sevoflurane-induced unconsciousness. Compared with baseline, couplings between the phase of IMF5 and the amplitude of IMF4 as well as the phase of IMF4 and the amplitude of IMF3 represent different coupling phases from the corresponding coupling at baseline. The coupling between the phase of IMF2 and the amplitude of IMF1 in anesthetic-induced unconsciousness differs from that at baseline and in sevoflurane-induced unconsciousness. The difference between the coupling phases of the two groups can be identified on the basis of the coupling between the phase of IMF6 and the amplitude of IMF5 in anesthetic-induced unconsciousness. The coupling phase is close to 3π/2 for Group A, but the amplitude distribution is similar to a uniform distribution for Group B.

## Discussion

In the present study, we determined changes in the coupling phase in both sevoflurane- and anesthetic-induced unconsciousness that were significantly qualitative. Decomposition using EMD was an advantage for this study because it is considered a superior method of decomposing nonlinear and nonstationary signals. Most relevant studies on PAC analysis have focused on the coupling between two specific cross-frequency bands. Linear and digital band-pass filters are used to extract the components with predetermined frequency bands. However, power leakage is a critical issue for decomposing a specific frequency-band component from a nonlinear and nonstationary signal using a linear filter. Furthermore, how to define appropriate frequency bands is a crucial topic for determining CFC. Different definitions lead to different results and conclusions in cross-frequency analysis. In this study, EMD was considered to reserve the properties of nonlinearity and nonstationary in decomposed IMFs and determine intrinsic frequency bands that are adaptive to the nature of signals. How to define appropriate frequency bands is no longer an issue. Furthermore, IMFs are superior components to those decomposed by band-pass filters using predetermined frequency bands for representing intrinsic dynamic characteristics on multiple intrinsic timescales.

EMD decomposes a signal into a set of IMFs that represents dynamic oscillations on multiple intrinsic timescales. This is helpful for taking a systematic approach in cross-timescale coupling analysis, such as the analysis results shown in Figs 2–5. According to the analysis results shown in Figs 2–4, the five couplings between the pairs of two successive IMFs represent significant couplings on multiple intrinsic frequency bands. To distinguish the unconsciousness of both sevoflurane- and anesthetic-induced from consciousness, the coupling phase between the phase of IMF4 and the amplitude of IMF3 is a critical marker. Moreover, the MI of the coupling between the phase of IMF4 and the amplitude of IMF3 represents the depth of anesthesia during the transitional process from consciousness through sevoflurane-induced unconsciousness to anesthetic-induced unconsciousness. The other three MIs for the couplings between the phase of IMF3 and the amplitude of IMf4, the phase of IMF5 and the amplitude of IMF4, and the phase of IMF6 and the amplitude of IMF5 are also appropriate indicators for assessing the depth of anesthesia.

**Figure 7:**
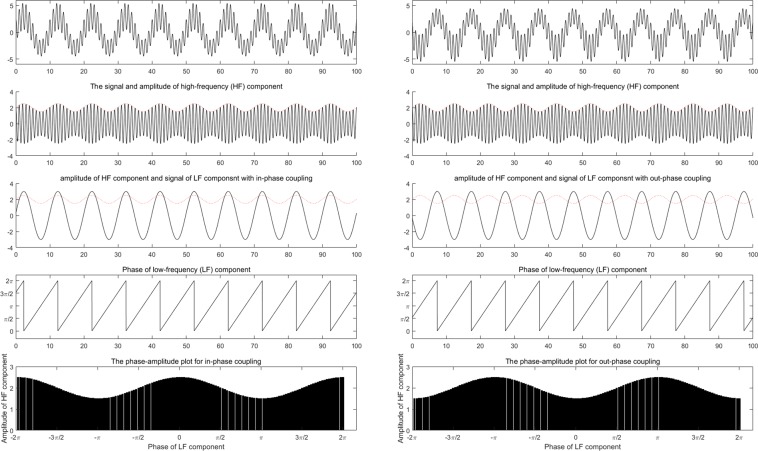
Illustrations for in-phase (left column) and out-phase (right column) PAC couplings.

In addition to distinguishing differences in the brain state affected by different anesthetic administrations with or without ketamine, the MI of the coupling between the phase of IMF2 and the amplitude of IMF1 is a more effective assessment compared with others. To illustrate different coupling phases found in the couplings between the phase of IMF4 and the amplitude of IMF3 for consciousness and unconsciousness, respectively, a numerical simulation was conducted to generate a high-frequency component modulated by a low-frequency oscillation, in which the phase difference between the amplitude envelope of the high-frequency component and the low-frequency oscillation represented the coupling phase. Figure 7 demonstrates the simulations of an in-phase and an out-phase PAC coupling. The left subfigure on the middle row presents two components: the red one is the amplitude of the high-frequency component and the black one is the low-frequency oscillation. The phase difference between the red and black is zero for an in-phase coupling. The right subfigure on the middle row presents another case of an out-phase coupling. The coupling phase of an out-phase coupling is π. According to the results of the simulation, the PAC coupling between the phase of IMF4 and the amplitude of IMF3 is similar to in-phase coupling in awareness and becomes an out-phase coupling in both sevoflurane- and anesthetic-induced unconsciousness. Moreover, the phase-amplitude of the coupling between the phase of IMF2 and the amplitude of IMF1 is similar to an out-phase PAC coupling in anesthetic-induced unconsciousness. These interesting characteristics reflect the qualitative profiles of anesthesia EEG in GA.

In conclusion, a multi-timescale approach to PAC analysis was proposed in this EMD-based study. Without the limitations of using predetermined frequency bands and the problem of power leakage caused by linear filters, the CFC analysis can be used to assess PAC couplings on multiple intrinsic timescales. The MI of the PAC coupling between the phase of IMF4 and the amplitude of IMF3 seems to be an indicator associated with the depth of anesthesia. According to the definitions of frequency bands for EEG signals, the PAC coupling between the phase of IMF4 and the amplitude of IMF3 represents a PAC coupling similar to the PAC between high-delta-band phase and theta-band amplitude. This finding matches the results reported in relevant studies, which reported that the weak coupling between the slow-wave phase and theta-band amplitude is a characteristic of anesthetic-induced unconsciousness. However, more details can be assessed in the multi-timescale approach based on EMD. We suggest that qualitative profiles (i.e., coupling phase) are as critical as quantitative results using the MI in the application of CFC analysis in anesthesia.

## Methods

### Patients

Sixty patients with low anesthetic risk (ASA I-II) were included in this study. Prior to surgical operation, the standard equipment for vital signs, including oximetry, ECG, BP, and a BIS monitor, were established prior to the induction of anesthesia. Sevoflurane induction with target control infusion began after monitoring patients for 1 minute for preanesthesia baseline measurements. Half of the included participants (Group A) were administered with ketamine with alfentanil, whereas the other half (Group B) were administered only alfentanil. When an anesthesiologist confirmed that the patients were losing consciousness, they received different regimens according to the specifications of their assigned group. Surgical operation began 5 minutes after regimen completion.

#### EEG recordings

In this study, EEG signals were recorded by the bispectral index (BIS) module of Philips MP60. A standard bifrontal montage was used to measure the frontal surface EEG at a sampling rate of 128 Hz.

### Ethics statements

This observational case–control study was approved by the Institutional Review Board of National Taiwan University Hospital (201508007RINB and 201509018RINB).

### Statement of informed consent

All methods were performed in accordance with relevant guidelines/regulations. All participants provided written informed consent after careful discussion. There are no conflicts of interest to report regarding this study.

### EMD

EMD is an innovative method used to decompose a nonlinear and nonstationary time series into a set of IMFs through a sifting process^21^. A sifting process derives the upper and lower envelopes of a given time series through cubic splines that connect the local maxima and minima separately. The final step of the sifting process removes the mean of the upper and lower envelopes from the given time series. The remaining part of the sifting process is an oscillatory component that is a candidate for IMF. The sifting process should be repeated until the oscillatory component satisfies two conditions: (1) for the entire oscillatory component, the difference between the numbers of extrema and zero-crossing must equal zero or one and (2) at any data point of the oscillatory component, the mean value of the upper and lower envelopes is zero. The oscillatory component that satisfies these two conditions is called an IMF and is denoted as *C*_*k*_. The difference between a time series and the *k-*th IMF is the *k-*th residual, which is denoted as *r*_*k*_, and is treated as data for decomposing the next IMF. The decomposition process should be repeated until the residual becomes monotonic or until only one extremum remains. The original data, *X*(*t*), can be reconstructed by the summation of *n* IMFs and the *n-*th residual:

1 $$X(t)=\sum _{k=1}^{n}{C}_{k}+{r}_{n}$$

### Ensemble empirical mode decomposition

In this study, an EEG signal was decomposed into a set of IMFs using ensemble EMD (EEMD)^20^, which is an enhanced algorithm based on the original EMD^21^. EEMD can solve the problem of mode-mixing in mode decomposition and obtain pure IMFs, and EMD functions as a dyadic filter bank^22^. Therefore, we can map the intrinsic frequency bands to the defined frequency bands for an EEG spectrum.

### PAC analysis

In PAC analysis, the phase of a low-frequency component and the amplitude of a high-frequency rhythm must be obtained. The time series of phases of a low-frequency component [denoted as ${\varnothing }_{{f}_{p}}(t)$] is obtained using Hilbert transform. Hilbert transform is also applied to extract the amplitude envelop of the high-frequency component [denoted as ${A}_{{f}_{A}}(t)$]. The composite time series $[{\varnothing }_{{f}_{p}}(t),{A}_{{f}_{A}}(t)]$ provides the amplitude of the high-frequency (*f*_*A*_) oscillation at each phase of low-frequency (*f*_*p*_) component. Next, the phases ${\varnothing }_{{f}_{p}}$ are binned, and the mean of ${A}_{{f}_{A}}$ over each phase bin is calculated. For each phase bin *j*, the mean amplitude is denoted as $< {A}_{{f}_{A}}{ > }_{{\varnothing }_{{f}_{p}}}(j)$. Lastly, the mean amplitude at each phase bin is normalized by dividing each bin value by the sum over the bins.

2 $$P(j)=\frac{ < {A}_{{f}_{A}}{ > }_{{\varnothing }_{{f}_{p}}}(j)}{{\sum }_{k=1}^{N} < {A}_{{f}_{A}}{ > }_{{\varnothing }_{{f}_{p}}}(k)}$$

where *N* is the number of phase bins, and *P* is the normalized amplitude for a phase bin. The normalized amplitude possesses the same characteristics as a discrete probability function (*pdf)*. Adriano *et al*. defined the MI based on KL distance as the PAC measure for representing the deviation of distribution *P* from the uniform distribution *U* (23). The KL distance (*D*_*KL*_) is related to the Shannon entropy and is calculated by

3 $${D}_{KL}(P,U)=\,\mathrm{log}(N)+\sum _{j=1}^{N}P(j)\mathrm{log}[P(j)]$$

The MI is defined by dividing the KL distance of observed amplitude distribution (P) from the uniform distribution (U) by log (N)

4 $$MI=\frac{{D}_{KL}(P,U)}{\mathrm{log}(N)}$$

### PAC analysis for multiple timescales

In this work, a multi-timescale approach of phase-amplitude coupling (PAC) analysis is based on the method of adaptive data decomposition by empirical mode decomposition (EMD). Then, multiple couplings between phases of low-frequency components and amplitudes of high-frequency components can be conducted to a segment of EEG data in a sequence. Here, the components decomposed by EMD are named as intrinsic mode functions (IMFs). The steps of multi-timescale PAC analysis are shown as follows:

1. Decompose an EEG signal into a set of IMFs.
2. Derive the time series of amplitude for IMF 1 as the amplitude of high-frequency component.
3. Derive the time series of phases of IMF 2–6 as phases of low-frequency components.
4. Calculate the modulation indexes (MI) for 5 couplings between phases of IMF2–6 and amplitude of IMF1.
5. Repeat steps 3–4 to calculate MI and phase-amplitude plots for the other 10 couplings between phases of IMF3–6 & amplitudes of IMF2, phases of IMF4–6 & amplitude of IMF3, phases of IMF5–6 & amplitude of IMF4, and phase of IMF6 & amplitude of IMF5.

### Statistical analysis

In this study, we conducted three statistical tests. The first one is the analysis of variance (ANOVA) for testing the intra-group difference among four stages of baseline, induction, the first 5 minutes in anesthesia, and the stage in general anesthesia. The second is the Kolmogorov-Smirnov test (KS test) for testing the paired difference between two groups on the same stage. The third test assesses the intra-group statistical differences between baseline and the other 3 stages using the KS test.
